# Inauguration of the Jenner Monument

**Published:** 1858-07

**Authors:** 


Inauguration of the Jenner Monument.—We see from the *Medical Times and Gazette* and the *Lancet*, that on Monday afternoon, the 16th of May, a meeting of some of the nobility and most eminent men in the medical profession, was held in the library of the College of Physicians, to inaugurate the statue of this eminent and immortal man. The attendance was large, and included about three hundred medical gentlemen. Prince Albert presided, and, on taking the chair, delivered a short and appropriate address, in which he eulogized Jenner as a great philosopher and philanthropist, as having done more to save human lives than any man of any age. Several other gentlemen addressed the meeting, and the Honorary Secretary, Mr. G. V. Irving, read the report of the Committee, in which special reference was made to the contributions to the fund from other countries. "America, with her usual energy, early took the lead, and chiefly through the zealous activity of Drs. Jackson, Ware, and Warren, of Boston; and those of Drs. Dunglison, Mütter, and Wood, of Philadelphia, speedily transmitted subscriptions to a large amount." The meeting terminated by passing resolutions, including votes of thanks to foreign nations for their support in the movement, as well as to the sculptor, Calder Marshall, R. A., the Committee, the Honorary Secretary, and the Chairman. The statue is made of bronze, and represents Jenner seated in a chair, in an easy position. The likeness is admirable, and the expression is that of a man in a thoughtful mood. It is placed in Trafalgar-Square, on the right of the statue recently erected to Sir Charles Napier.

